# miR-1262 suppresses gastric cardia adenocarcinoma via targeting oncogene *ULK1*

**DOI:** 10.7150/jca.46971

**Published:** 2021-01-01

**Authors:** Yan Zheng, Mengyu Xie, Nasha Zhang, Jiandong Liu, Yemei Song, Liqing Zhou, Ming Yang

**Affiliations:** 1 Research Center of Translational Medicine, Jinan Central Hospital Affiliated to Shandong First Medical University, Jinan, Shandong, China.; 2 Shandong Provincial Key Laboratory of Radiation Oncology, Cancer Research Center, Shandong Cancer Hospital and Institute, Shandong First Medical University and Shandong Academy of Medical Sciences, Jinan, Shandong Province, China.; 3 Jinan Central Hospital, Cheeloo College of Medicine, Shandong University, Jinan, Shandong, China.; 4 Cheeloo College of Medicine, Shandong University, Jinan, Shandong, China.; 5 Department of Radiation Oncology, Huaian No. 2 Hospital, Huaian, Jiangsu, China.

**Keywords:** miR-1262, gastric cardia adenocarcinoma, tumor suppressor, genetic polymorphism, ULK1

## Abstract

Gastric cardia adenocarcinoma (GCA) is one of two main gastric cancer subtypes and has its own epidemiological, pathogenic and clinical characteristics. Genetic polymorphisms locating in a microRNA (miRNA) gene enhancer could transcriptionally regulates miRNA expression via impacting binding of transcriptional factors. It is still unclear how miR-1262 and a potential regulatory rs12740674 polymorphism mapping to a strong enhancer region of *miR-1262* contribute to GCA development. We genotyped *miR-1262* rs12740674 in two independent case-control sets consisting of 1,024 GCA patients and 1,118 controls, and found that the rs12740674 CT or TT genotype carriers had a 0.69-fold decreased risk to develop GCA compared to the CC genotype carriers (95% confidence interval=0.57-0.84, *P*=2.1×10^-4^). In the genotype-phenotype correlation analyses of 21 pairs of GCA-normal tissues, the rs12740674 CT or TT genotype was associated with significantly increased levels of miR-1262. Cell proliferation, wound healing and transwell assays elucidated that miR-1262 is a novel GCA tumor suppressor. Consistently, a significantly down-regulated level of miR-1262 exists in GCA specimens compared to normal tissues. Furthermore, multiple lines of evidences indicated that oncogene *ULK1* is the target gene of miR-1262 in GCA. Our findings demonstrate miR-1262 transcriptionally modulated by an enhancer genetic variant suppresses GCA via targeting oncogene *ULK1*. Our data highlight miR-1262 as a promising diagnostic marker and therapeutic target for GCA.

## Introduction

Gastric cancer is a common malignancy of the gastrointestinal system and has been one of the leading causes of cancer death over the past several decades 1. As the second most common cancer and the third most common cause of cancer death in China, gastric cancer was accounting for about 11.6% of all new cancer cases 2. Gastric cardia carcinoma (GCA) and non-cardia carcinoma are two major gastric cancer subtypes. In 2012, the age standardized incidence rates of GCA were 3.3 per 100,000 worldwide 3. GCA shows distinctive epidemiological characteristics, etiology, pathogenesis and clinical manifestations compared to non-cardia gastric carcinoma 4. There are multiple known risk factors for GCA, such as cigarette smoking, heavy alcohol consumption, low-intake of fruits and vegetables, dietary carcinogen exposure and gastroesophageal reflux disease 5-8. Accumulating evidences including genome-wide association studies (GWAS) of gastric cancer demonstrated that some common genetic variants are significantly associated with GCA development 9-13. However, these loci only contribute to a small proportion of GCA predisposition, elucidating that additional GCA-susceptibility genetic variants need to be identified.

As a highly conserved small noncoding single-stranded RNAs from plants to humans, microRNAs (miRNA) act as a new class of gene expression regulators 14. It has been reported that miRNA alterations are involved in the initiation and progression of human cancers 15. Genes encoding miRNAs frequently acquire chromosome gain or a loss and result in dysregulated functions of miRNAs in cancers, which marking them as promising therapeutic targets 15,16. Besides changes of cancer-associated genomic regions, differential expression of miRNAs in the tumor tissue relative to the normal adjacent tissue can be the consequence of germline genetic variations 16,17. Xie et al. systematically annotated miRNA-related promoter single nucleotide polymorphisms (SNPs) and enhancer SNPs using the Encyclopedia of DNA Elements (ENCODE) data. They found that the SNP rs12740674 is located at 61,743bp downstream of *miR-1262* and maps to a strong enhancer region. Interestingly, the rs12740674 T allele was associated with reduced miR-1262 expression in lung tissues. Additionally, miR-1262 could significantly inhibit lung cancer cell proliferation 18.

In this study, we firstly evaluated the role of *miR-1262* in GCA. We found that miR-1262 is evidently down-regulated in GCA specimens compared to normal tissues. Further investigation in gastric adenocarcinoma and esophageal adenocarcinoma cell lines revealed that miR-1262 could inhibit cell proliferation, colony formation, cell cycle progression and cell invasion via directly targeting *ULK1*, an oncogene in adenocarcinoma cells. The significant association between *miR-1262* rs12740674 polymorphism and GCA risk was examined in two case-control sets. Our findings gain insights of the biological functions of miR-1262 and highlight the critical involvement of miR-1262 in GCA.

## Materials and methods

### Study case-control sets

Two case-control sets were enrolled in the current study (Table S1). (a) Shandong discovery set: 584 GCA patients and 568 matched controls [sex- and age-matched (±5 years)] from Shandong Cancer Hospital and Institute (Jinan, Shandong, China). The 568 cancer-free control subjects were randomly selected from a pool of 6000 individuals from a comprehensive physical examination conducted in Jinan city and the surrounding areas during the same time period as the patients were collected. (b) Jiangsu validation set: 440 GCA patients and 500 matched controls from Huaian No. 2 Hospital (Huaian, Jiangsu, China). Controls were cancer-free individuals selected from a community cancer-screening program (3000 individuals) for early detection of cancer conducted in Huaian city during the same time period as the patients were collected. All subjects were ethnic Han Chinese. The case-control sets have been reported previously 11. Twenty-one pairs of GCA specimens and normal tissues adjacent to the tumors were obtained from surgically removed specimens of GCA patients in Shandong Cancer Hospital and Institute. At recruitment, informed consent was obtained from each subject and each participant was then interviewed to collect detailed information on demographic characteristics, such as sex, age, cigarette smoking and alcohol drinking. This study was approved by the institutional Review Boards of Shandong Cancer Hospital and Institute and Huaian No. 2 Hospital.

### Polymorphism genotyping

Genotypes of *miR-1262* rs12740674 genetic polymorphism were detected using the iPLEX Sequenom Mass Array platform (Sequenom Inc., San Diego, CA, USA) as reported previously (Table S2) 19,20. A 15% blind, random sample of study subjects was genotyped in duplicates and the reproducibility was 100%. To confirm these results, Sanger sequencing of CC, CT and TT genotypes was also performed (Figure S1).

### Cell culture and reagents

Human gastric adenocarcinoma cell lines MGC80-3 and HGC-27 were provided by Dr. Yunshan Wang (Jinan Central Hospital Affiliated to Shandong University, Jinan, China). They were tested and authenticated by short tandem repeat profiling (Jinan Dean Forensic Identification Institute, Jinan, China) in September 26, 2019. Esophageal adenocarcinoma cell line OE33 was purchased from JENNIO Bio-Technology Company (Guangzhou, China), and authenticated using short tandem repeat analysis by JENNIO Biotech on May 8, 2018. Human MGC80-3, HGC-27 and OE33 cells were cultured at 37 °C with 5% CO2 using DMEM or RPMI-1640 media with 10% fetal bovine serum (Hyclone). Genotypes of MGC80-3, HGC-27 and OE33 cells were identified by Sanger sequencing. Mimics and inhibitors of miR-1262, siRNAs of ULK1 or the negative control RNA duplex (NC) which was nonhomologous to any human genome sequence were synthesized by Genepharma (Shanghai, China). Small RNAs were transfected into MGC80-3, HGC-27 and OE33 cells with INTERFERin® (Polyplus). Antibodies against ULK1 (abcam, ab167139) and β-Actin (abcam, ab8226) were used.

### Quantitative reverse transcription PCR (qRT-PCR)

Total RNA was isolated from GCA tissues, normal tissues as well as MGC80-3, HGC-27 and OE33 cells with Trizol (Invitrogen). Each RNA sample was treated with RNase-Free DNase (Invitrogen) to avoid contamination of genomic DNA. The corresponding cDNAs were generated using the Revert Ace kit (TOYOBO, Osaka, Japan). As described previously 19, human miR-1262 and U6 small RNA were detected with their specific stem-loop RT-PCR primers (Ribobio, Guangzhou, China) using SYBR-Green qRT-PCR. *ULK1* and* β-actin* mRNA expression was examined using their specific qRT-PCR primers (Table S3).

### miR-1262 and ULK1 reporter gene constructs

We cloned a potential 491bp enhancer region around the rs12740674 genetic polymorphism from human genomic DNA using specific primer pairs with *Xho*I and *Kpn*I restriction sites (Table S3). The PCR products and the pGL3-Promoter vector (Promega) were digested with *Xho*I and *Kpn*I (TaKaRa). The digested PCR products were then ligated into the pGL3-Promoter vector. The resultant two plasmids were designated as p-C or p-T. The orientation and integrity of these constructs were confirmed through Sanger sequencing.

The sequence corresponding to the wild-type *ULK1* 3'-UTR (3916-4114nt) was amplified with MGC80-3 cDNA using Pyrobest^TM^ DNA Polymerase (TaKaRa) (PCR primers shown in Table S3). The PCR products with blunt ends were ligated into the appropriately digested pGL3-Promoter. The resultant plasmid, designated as ULK1-WT, was sequenced to confirm the orientation and integrity. The *ULK1* reporter gene plasmid with mutant miR-1262 binding site was constructed with QuikChange Site-Directed Mutagenesis kit (Stratagene, La Jolla, CA). These mutant plasmids were confirmed by DNA sequencing and named as ULK1-MU.

### Dual luciferase reporter assays

For rs12740674 reporter assays, reporter constructs (pGL3-Promoter, p-C and p-T) were transfected into MGC80-3, HGC-27 and OE33 cells. For *ULK1* reporter assays, pGL3-Promoter, ULK1-WT, and ULK1-MU reporter constructs plus 20nmol/L miR-1262 mimics were transfected into MGC80-3, HGC-27 and OE33 cells. pRL-SV40 (1 ng) (Promega) containing renilla reniformis luciferase was cotransfected to standardize transfection efficiency. Luciferase activities were detected at 48h after transfection using a luciferase assay system (Promega). For each luciferase construct, three independent transfections were done (each in triplicate).

### Cell proliferation, apoptosis and cell cycle analyses

Human MGC80-3, HGC-27 and OE33 cells were seeded in a 12 well plate and transfected with 10nM miR-1262 mimics, miR-1262 inhibitors, *ULK1* siRNAs or NC RNA (Genepharma) with INTERFERin^®^ (Polyplus). Cells were harvested and counted at 24h, 48h and 72h after transfection. Apoptosis of transfected MGC80-3, HGC-27 and OE33 cells was determined using the Alexa Fluor 488 annexin V/Dead Cell Apoptosis Kit (Invitrogen) with FACS Calibur flow cytometer (FCM) (BD Biosciences). For cell cycle analyses, transfected MGC80-3, HGC-27 and OE33 cells were dyed with PI and detected with the FACS Calibur FCM.

### Colony formation assays

A total of 2,000 MGC80-3 cells, 2,000 HGC-27 cells or 3,500 OE33 cells were seeded into a 6-well cell culture plate. All cells were transfected with 10nM miR-1262 mimics, miR-1262 inhibitors, *ULK1* siRNAs or NC RNA. After 12 days, cells were washed with cold PBS twice, permeated with methyl and fixed with 3.7% formaldehyde. MGC80-3, HGC-27 or OE33 cells were dyed with crystal violet. Colony number in each well was counted under microscopy.

### Wound healing and transwell assays

When the cell layer of MGC80-3, HGC-27 or OE33 transfected with miR-1262 mimics, miR-1262 inhibitors, *ULK1* siRNAs or NC RNA reached about 90% confluence, a wound was scratched by a 10 μl pipette tip during wound healing assays. The average extent of wound closure was measured after MGC80-3, HGC-27 or OE33 cells were continued cultured at 37 °C. During transwell assays, MGC80-3, HGC-27 or OE33 transfected with miR-1262 mimics or NC RNA were added to upper transwell chambers (pore 8 mm, Corning) that were coated with 100 mL BD Matrigel overnight. A total of 650 μl culture medium containing 10% FBS was added to the lower wells. After incubation for 48 h, MGC80-3, HGC-27 or OE33 cells in transwell chambers were fixed and stained. The nonmigratory cells were scraped from the upper part of the filter and cells migrated to the lower wells through pores were stained with 0.2% crystal violet solution and counted.

### Statistics

The difference in demographic variables, smoking status and drinking status was calculated using Pearson's χ^2^ test. The associations of *miR-1262* rs12740674 genotypes and GCA risk were examined by ORs and their 95% CIs computed by logistic regression models. All ORs were adjusted for age, sex, smoking or drinking status, where it was appropriate. Student's *t* test or one-way analysis of variance was used to comparing differences between groups. The significance of expression association between *miR-1262* and *ULK1* mRNA was examined by Spearman's correlation. All data shown are representative of three independent experiments, and each experiment was analyzed independently. A *P* value of less than 0.05 was used as the criterion of statistical significance. All analyses were performed with SPSS16.0 (SPSS Inc.) or GraphPad Prism (Version 5, GraphPad Software, Inc.).

## Results

### Association between miR-1262 rs12740674 polymorphism and GCA risk

Associations between genotypes of *miR-1262* rs12740674 genetic polymorphism and GCA risk was calculated using unconditional logistic regression analyses (Table 1). In Shandong set, the rs12740674 T allele was found to be a protective allele; subjects carrying the CT or TT genotype had an OR of 0.71 (95%CI=0.55-0.92, *P*=0.011) for developing GCA, compared with subjects having the CC genotype. The OR of having the rs12740674 CT genotype in patients was 0.67 (95%CI=0.51-0.89, *P*=0.005) compared with the CC genotype. In Jiangsu validation set, the significant association between *miR-1262* rs12740674 polymorphism and GCA risk was also observed. Carriers of rs12740674 CT genotype showed significantly decreased risk to develop GCA compared with the CC genotype carriers (OR=0.67, 95%CI=0.50-0.90, *P*=0.008) (Table 1). Similarly, individuals with the CT or TT genotype also showed significantly decreased GCA risk compared with those with the CC genotype in the validation set (OR=0.67, 95%CI=0.50-0.89, *P*=0.006). In the pooled analyses, we found that the rs12740674 CT or TT genotype carriers had a 0.69-fold decreased risk to develop GCA compared to the CC genotype carriers (95% CI=0.57-0.84, *P*=2.1×10^-4^) (Table 1). Logistic regression analyses also revealed that the rs12740674 CT genotype was significantly associated with a decreased risk of GCA (OR=0.67, 95%CI=0.55-0.82, *P*=1.1×10^-4^), although the association between the TT genotype and GCA susceptibility was not statistically significant (*P*>0.05). Our data showed significant association between the rs12740674 SNP and GCA in Chinese populations.

### Stratified analyses of association between the miR-1262 polymorphism and GCA risk

The risk of GCA associated with the *miR-1262* rs12740674 genetic variant was further evaluated by stratifying for age, sex, smoking and alcohol drinking status using the combined data of two case-control sets (Table 2). The middle age of healthy controls is 66 years old and, thus, we used age 66 for stratification. Compared with the CC genotype, a decreased risk of GCA was only associated with rs12740674 CT or TT genotype for the group aged 66 years or younger (OR=0.60, 95%CI=0.46-0.79,* P*=2.6×10^-4^), but not in the group aged older than 66 years (OR=0.79, 95%CI=0.60-1.04, *P*=0.097). There was statistically significant gene-age interaction (*P*_interaction_=0.039). A significantly reduced GCA risk associated with the rs12740674 CT or TT genotype compared with the CC genotype was observed for both males (OR=0.74, 95%CI=0.60-0.92, *P*=0.006) or females (OR=0.46, 95%CI=0.27-0.78, *P*=0.004). In stratified analyses with smoking or alcohol drinking status, the rs12740674 genetic polymorphism was significantly associated with decreased risk in either non-smokers (OR=0.58, 95%CI=0.45-0.74, *P*=1.4×10^-5^) or non-drinkers (OR=0.59, 95%CI=0.47-0.75, *P*=1.6×10^-5^), but not in smokers (OR=0.96, 95%CI=0.66-1.39, *P*=0.817) or drinkers (OR=0.97, 95%CI=0.65-1.44, *P*=0.884). A statistically significant gene-smoking or gene-drinking interaction was observed (*P*_interactioin_=0.041 or 0.018). That is, smoking and alcohol drinking might increase GCA susceptibility among individuals carrying the risk genotypes. Associations between rs12740674 and GCA risk stratified by disease stages were also analyzed (Table S2).

### Decreased miR-1262 expression in GCA and its association with rs12740674 genotypes

**Figure 1:**
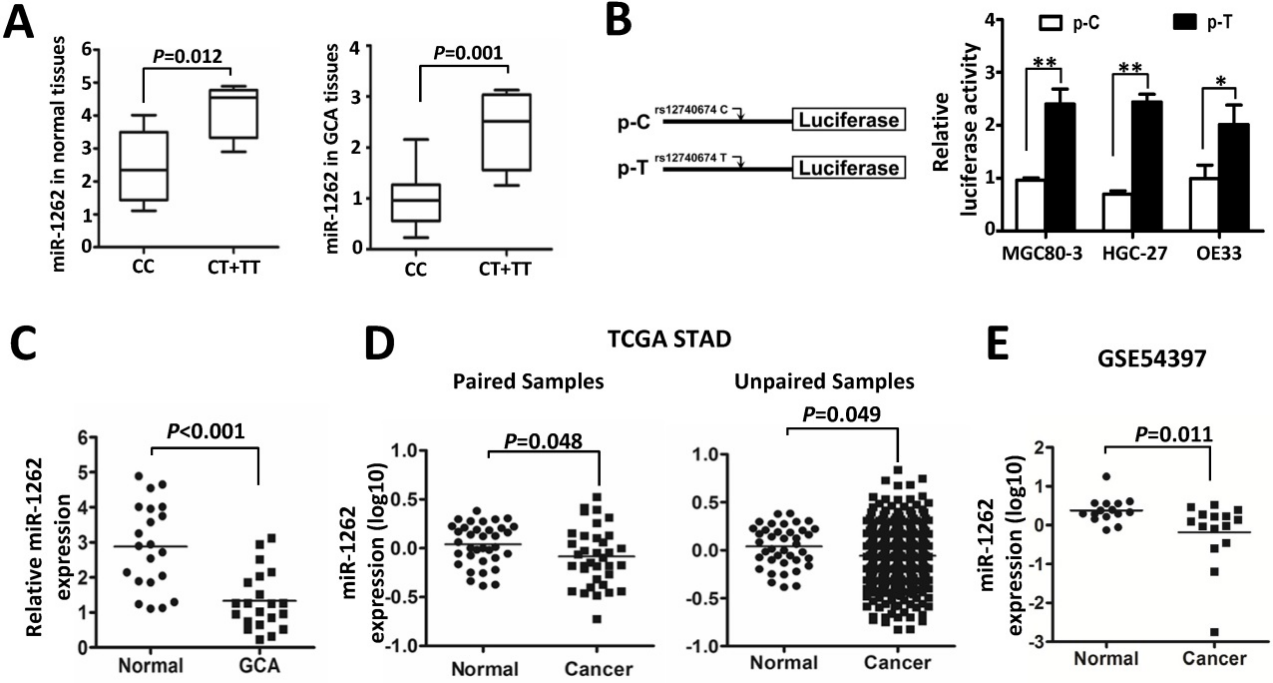
MiR-1262 expression in tissue specimens and its allelic regulation. (A) miR-1262 was quantified using qRT-PCR in 21 GCA-normal pairs. All data of miR-1262 expression were normalized to U6 expression levels. miR-1262 expression in GCA and normal tissues grouped by rs12740674C>T genotypes. (B) Transient luciferase reporter gene expression assays with constructs containing different rs12740674 alleles in MGC80-3, HGC-27 and OE33 cells. pRL-SV40 were cotransfected with these constructs to standardize transfection efficiency. Fold-changes were detected by defining the luciferase activity of cells co-transfected with pGL3-promoter as 1. (C) Increased miR-1262 expression in GCA tissues compared to normal tissues. (D) miR-1262 expression in TCGA stomach adenocarcinoma (STAD) compared with the normal tissues. (E) miR-1262 expression in GSE54397.

The *miR-1262* rs12740674 C>T genetic variant locating in a potential gene enhancer region, may lead to differential binding of multiple tansfactors, i.e. FOS, and dysregulatory expression of miR-1262 in cancer cells 18. Therefore, we firstly examined whether there is an allele-specific effect of the rs12740674 SNP on miR-1262 expression in paired GCA and normal tissues. As shown in Figure 1A, we found that subjects with the rs12740674 CC genotype had significantly lower miR-1262 levels (mean ± SE) than those with the CT and TT genotypes in either GCA tissues (1.018±0.138 [*n*=16] vs. 2.338±0.348 [*n*=5], *P*=0.001) or the normal gastric tissues (2.482 ± 0.263 [*n*=16] vs. 4.145 ± 0.366 [*n*=5], *P*=0.012) (Figure 1A). We then evaluated how rs12740674 genetic polymorphism impacts miR-1262 expression using dual-luciferase reporter gene assays in human MGC80-3, HGC-27 and OE33 cells (Figure 1B). The rs12740674 T allelic reporter construct (p-T) showed significantly higher luciferase activities compared to cells expressing the rs12740674 C allelic reporter construct (p-C) (all *P*<0.05). Taken together, these data indicate that the enhancer carrying the rs12740674 T allele could more efficiently promote *miR-1262* transcription compared to the rs12740674 C allelic enhancer.

In order to identify the role of miR-1262 during GCA development, we analyzed its expression in our cohort or datasets from TCGA or public available profiling data. As shown in Figure 1C, miR-1262 was significantly repressed in GCA tissues compared to normal tissues (*P*<0.001). Similarly, there is signifcantly down-regulated miR-1262 expression in stomach adenocarcinoma (STAD) compared with the normal tissues (Paired samples: *P*=0.048; unpaired samples: *P*=0.049) (Figure 1D). In line with our results and the TCGA data, we also observed evidently reduced miR-1262 expression in gastric cancer tissues compared to normal specimensin a cohort of Korean individuals (GSE54397) (*P*=0.011) (Figure 1E).

### MiR-1262 suppresses proliferation, migration and invasion of adenocarcinoma cells

**Figure 2:**
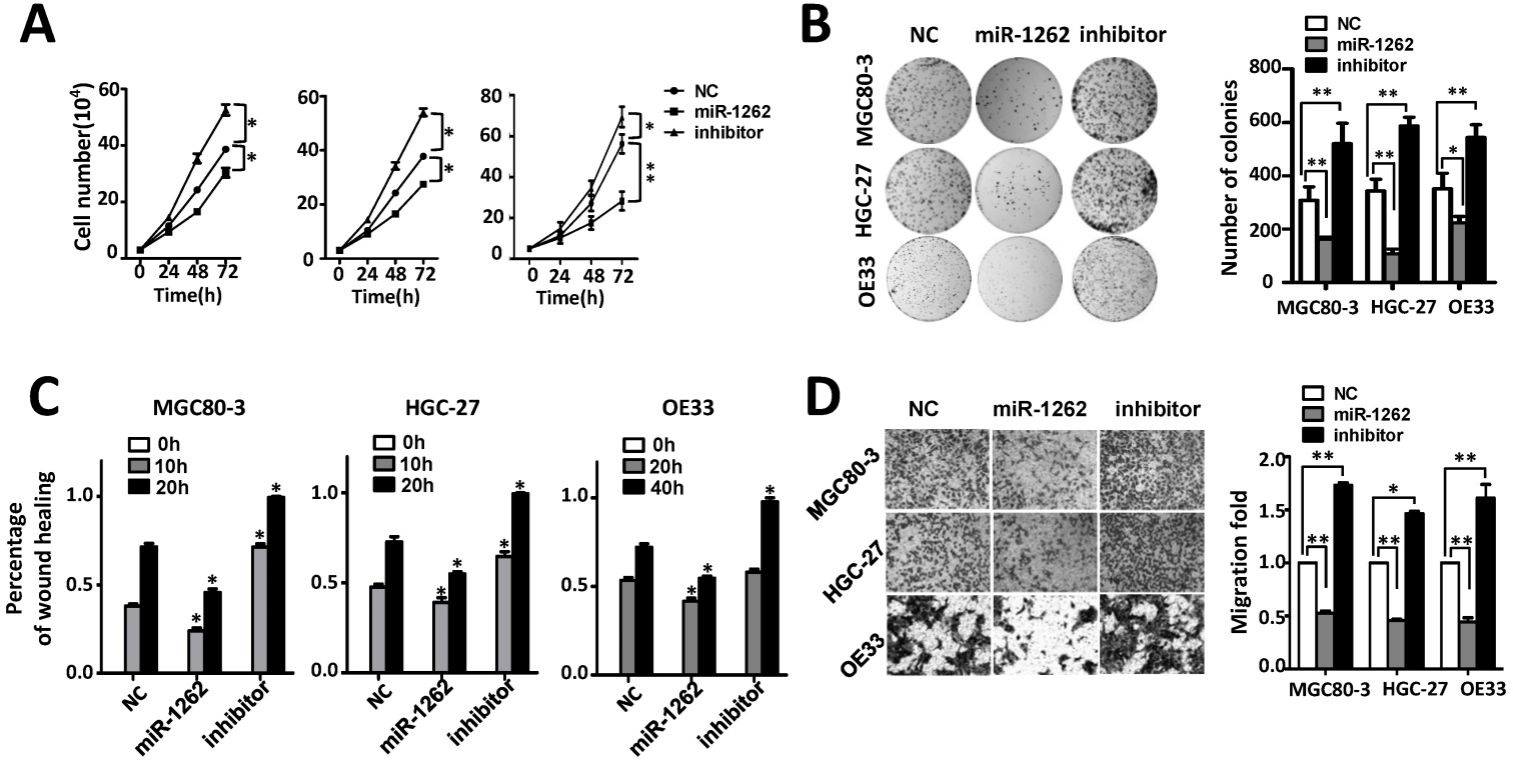
MiR-1262 inhibits cell proliferation, migration and invasion capability of MGC80-3, HGC-27 and OE33 adenocarcinoma cells. (A) miR-1262 inhibits cell growth of MGC80-3, HGC-27 and OE33 adenocarcinoma cells. Cell number was counted at 24 h, 48 h and 72 h after transfection. (B) Colony formation assays indicate that miR-1262 significantly suppresses colony formation of MGC80-3, HGC-27 and OE33 adenocarcinoma cells. (C) miR-1262 inhibits wound-healing in MGC80-3, HGC-27 and OE33 cells. Wound fields were observed directly after removal of inserts (0h) and cell migration was followed for 10 h or 20 h (MGC80-3 and HGC-27) and 20 h or 40 h (OE33). (D) miR-1262 suppresses invasion ability of MGC80-3, HGC-27 and OE33 cells. Cells on the lower surface of the chamber were stained by crystal violet at 48 h after transfection. Cell counts data of MGC80-3, HGC-27 and OE33 cells were presented as histogram. All results of the mean of triplicate assays with standard deviation of the mean are presented. ^*^P< 0.05, ^**^P< 0.01.

We investigated how miR-1262 impacts malignant behaviors of human gastric adenocarcinoma MGC80-3 and HGC-27 cells as well as esophageal adenocarcinoma OE33 cells (Figure 2). As shown in Figure 2A, miR-1262 can significantly reduce proliferation of MGC80-3, HGC-27 and OE33 cells, indicating its tumor suppressor nature in GCA. Consistent to the cell proliferation assays, miR-1262 shows similarly inhibiting capability on colony formation of MGC80-3, HGC-27 and OE33 cells (all *P*<0.05) (Figure 2B). To gain insights into the functional relevance of miR-1262 in GCA proliferation, we examined how it impacts cell cycle progression and apoptosis of MGC80-3, HGC-27 and OE33 cells. Compared with NC RNA-transfected cells, miR-1262 could significantly elevated G2/M population of MGC80-3 cells (*P*<0.05). Similar results were also found in HGC-27 and OE33 cells (both *P*<0.05) (Figure S2). However, no significant miRNA-induced apoptosis was observed in all three cell lines (Figure S3).

It was also found that miR-1262 could significantly reduce the motility of MGC80-3, HGC-27 and OE33 cells compared to the control cells transfected with NC RNA (Figure 2C and Figure S4). In line with the wound-healing assays, impaired invasion capability was observed after elevated expression of miR-1262 (all *P*<0.001) (Figure 2D). On the contrary, inhibitors of miR-1262 are able to significantly increase motility and invasion capability of MGC80-3, HGC-27 and OE33 cells (all *P*<0.05). These data elucidate that miR-1262 acts as a tumor suppressor in GCA.

### Identification of ULK1 as a direct target of miR-1262 in GCA

**Figure 3:**
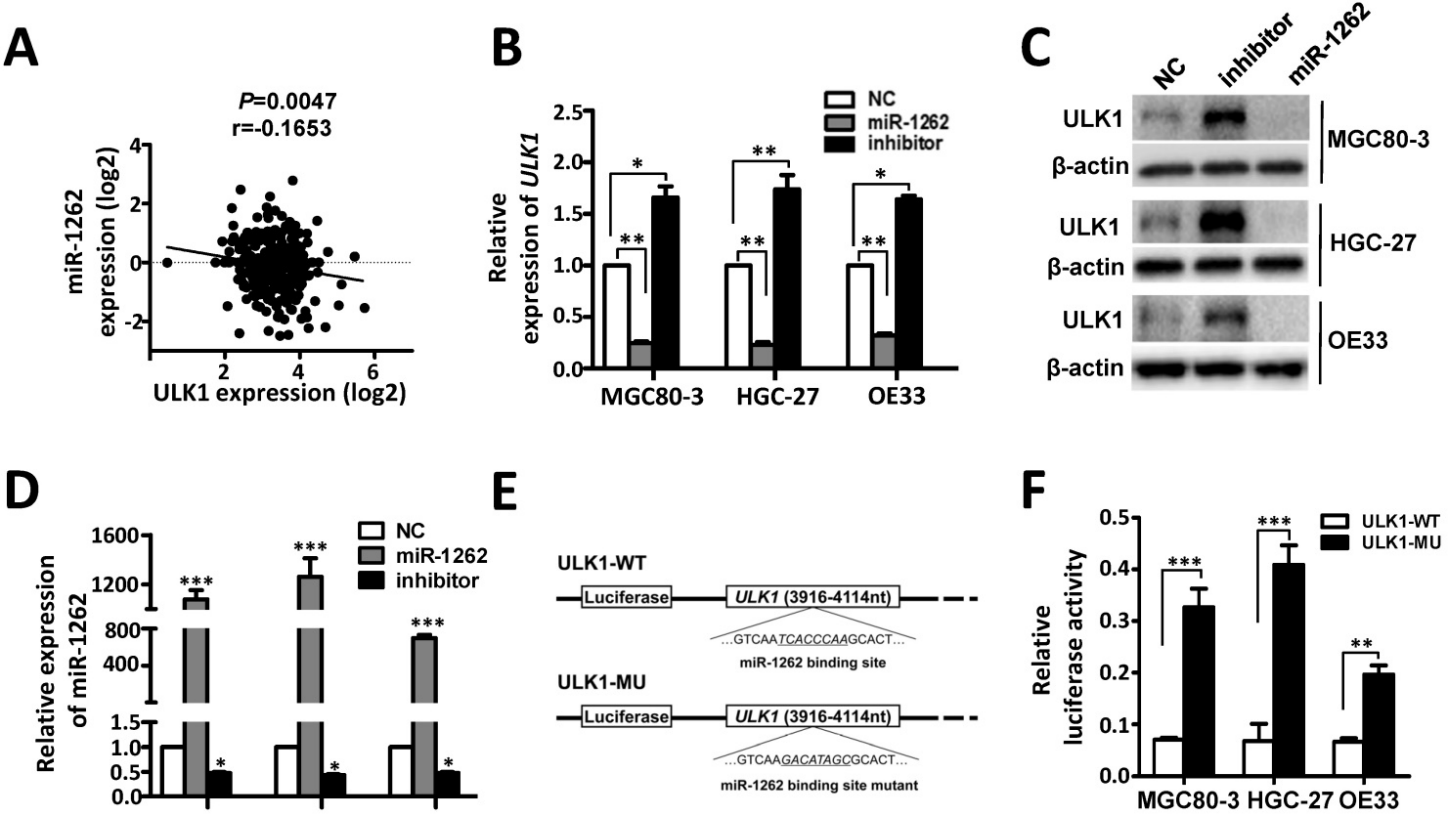
Identification of *ULK1* as a direct target of miR-1262 in GCA. (A) The negative expression correlation between *ULK1* mRNA and miR-1262 in gastric cancer tissues. (B) qRT-PCR validation of *ULK1* mRNA of miR-1262 in MGC80-3, HGC-27 and OE33 cells transfected with miR-1262 mimics, miR-1262 inhibitors or NC RNA. (C) miR-1262 could significantly inhibit ULK1 protein expression in MGC80-3, HGC-27 and OE33 cells. (D) Relative miR-1262 expression in MGC80-3, HGC-27 and OE33 cells. (E) Schematic constructions of ULK1-WT and ULK1-MU. (F) MGC80-3, HGC-27 and OE33 cells were co-transfected with ULK1-WT and miR-1262 mimics or ULK1-MU and miR-1262 mimics. Luciferase activity was detected at 48h after transfection and normalized relative to the Renilla luciferase expression. All results of the mean of triplicate assays with standard deviation of the mean are presented. ^*^P< 0.05, ^**^P< 0.01, ^***^P< 0.001.

*ULK1* was predicted to be a target gene of miR-1262 18. In support of this, we observed significantly negative expression correlation between *ULK1* mRNA and miR-1262 in gastric cancer tissues (Figure 3A). In MGC80-3, HGC-27 and OE33 cells, miR-1262 could evidently down-regulate mRNA and protein expression of *ULK1* (Figure 3B-3D). In line with this, inhibition of miR-1262 leads to obviously elevated *ULK1* expression in all three cell lines (Figure 3B-3D). Dual luciferase reporter gene assays were then conducted to examine the potential direct interaction between miR-1262 and the *ULK1* 3'UTR. We firstly subcloned a 199bp human *ULK1* 3'UTR sequence linked to the firefly luciferase gene and referred the construct to as ULK1-WT (Figure 3E). Point substitutions were introduced to ULK1-WT to disrupt the binding site of miR-1262 and the mutant construct was referred to as ULK1-MU (Figure 3E). MGC80-3, HGC-27 and OE33 cells were co-transfected with ULK1-WT and miR-1262 mimics or ULK1-MU and miR-1262 mimics. Significantly decreased luciferase activities were found in the ULK1-WT transfected group compared to that of the ULK1-MU group in MGC80-3, HGC-27 and OE33 cells (all *P*<0.01) (Figure 3F). Taken together, our results indicate that *ULK1* is a target gene of miR-1262 in GCA.

### *ULK1* acts as an oncogene in GCA

**Figure 4:**
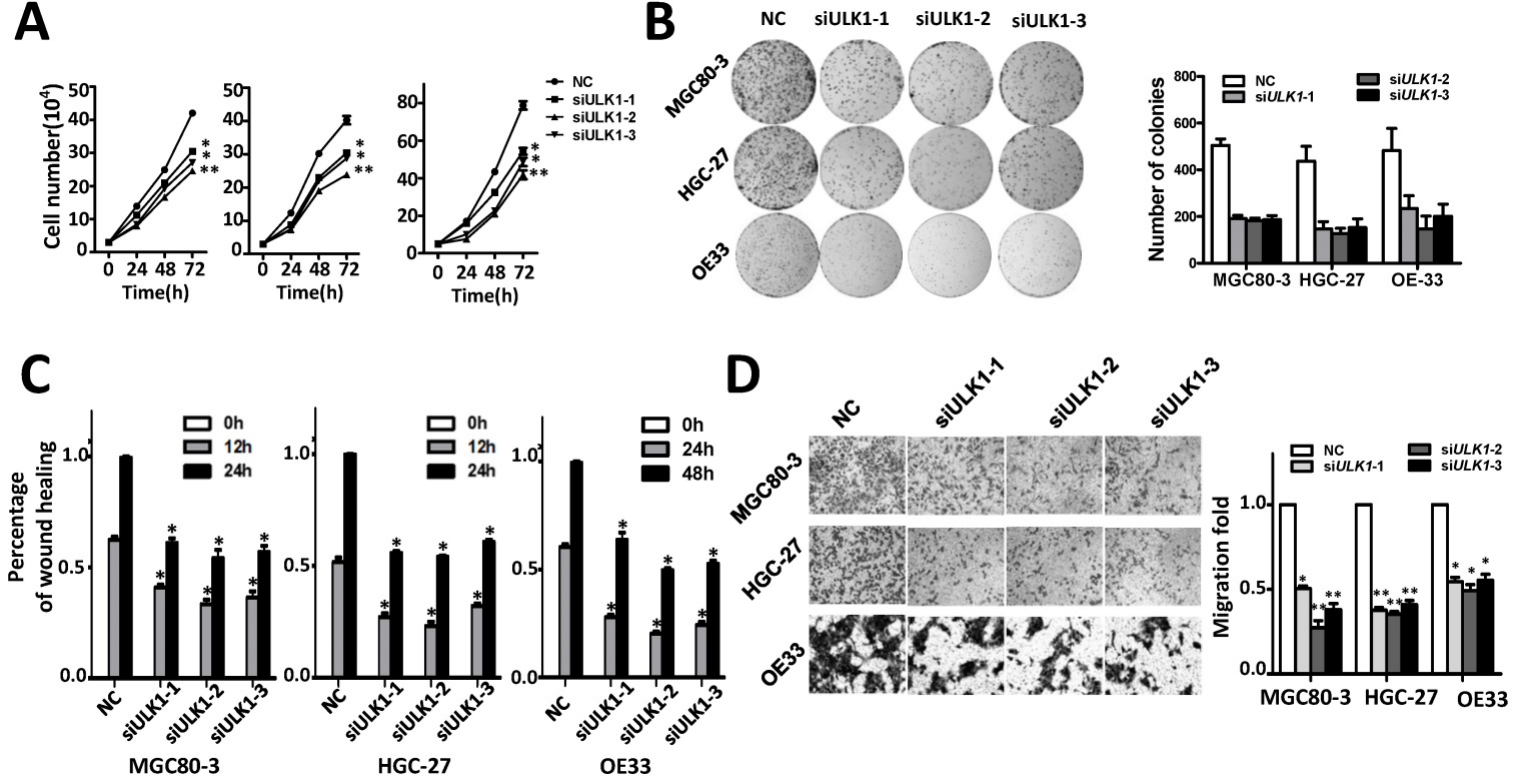
Silencing ULK1 inhibits cell proliferation migration and invasion capability of MGC80-3, HGC-27 and OE33 adenocarcinoma cells. (A) Silencing ULK1 inhibits cell growth of MGC80-3, HGC-27 and OE33 adenocarcinoma cells. Cell number was counted at 24 h, 48 h and 72 h after transfection. (B) Colony formation assays indicate that *ULK1* siRNAs significantly suppresses colony formation of MGC80-3, HGC-27 and OE33 adenocarcinoma cells. (C) *ULK1* siRNAs inhibit wound-healing in MGC80-3, HGC-27 and OE33 cells. Wound fields were observed directly after removal of inserts (0 h) and cell migration was followed for 12h or 24h (MGC80-3 and HGC-27) and 24 h or 48 h (OE33). (D) *ULK1* siRNAs suppress invasion ability of MGC80-3, HGC-27 and OE33 cells. Cell counts data of MGC80-3, HGC-27 and OE33 cells were presented as histogram. All results of the mean of triplicate assays with standard deviation of the mean are presented. *P< 0.05, **P< 0.01.

The oncogene functions of *ULK1* were determined in MGC80-3, HGC-27 and OE33 cells (Figure 4, Figure S5). Silencing ULK1 can significantly suppress proliferation of MGC80-3, HGC-27 and OE33 cells (Figure 4A). In line with this, various *ULK1* siRNAs evidently inhibited colony formation of these cells (Figure 4B). Moreover, *ULK1* are capable to accelerate migration and invasion of adenocarcinoma cells. As shown in Figure 4C-4D and Figure S6 silencing ULK1 could significantly reduce the motility and invasion capability of MGC80-3, HGC-27 and OE33 cells compared to the control groups.

## Discussion

Accumulated evidences demonstrated that genetic makeups contribute to etiology of GCA 9-13. Deregulation of miRNAs has been frequently observed in malignant tissues. We found that the rs12740674 genetic variation in the *miR-1262* enhancer can result in allelic, differential expression of miR-1262 in GCA. The *miR-1262* rs12740674 genetic polymorphism was significantly associated with GCA risk in Chinese populations. Moreover, we for the first time demonstrated that miR-1262 acts as a novel tumor suppressor via suppressing expression of oncogene *ULK1* in GCA.

In line with the role of miR-1262 in lung cancer, we also observed apparently reduced expression of miR-1262 in GCA tissues of our cohort as well as in gastric cancer tissues from TCGA and GEO datasets. Individuals with rs12740674 CT or TT genotypes showed significantly higher expression of miR-1262 in both GCA tissues and normal gastric tissues. Inconsistent to results in lung cancer, we found that rs12740674 T allele in the *miR-1262* enhancer element significantly decreased GCA risk, compared with the C allele. It is in line with the association between the rs12740674 polymorphism and decreased GCA risk since the protective T allele is associated with elevated expression of tumor suppressor miR-1262.

Others and we reported that expression of miRNAs could be fine-regulated by some crucial TFs 21-28. Interestingly, rs12740674 locating in a region of proteins bound, including FOS, MAFF, MAFK and EP300 18. Among these TFs, FOS is an important component of Activating Protein 1 (AP-1) and involved in transcriptional regulation of miRNAs, i.e. FOS binds to the miR-551a promoter, activates its expression and blocks tumorigenesis 25-28. HaploReg prediction indicated that the C-to-T change of rs12740674 remarkably increased the AP-1 motif binding affinity (from 1.4 to 12.2), which might subsequently induce miR-1262 expression in GCA.

In GCA, we found that *ULK1* was a target gene of miR-1262. ULK1 plays an important part in the initiation of autophagy 29. The activity of ULK1 kinase is positively regulated by the adenosine monophosphate-activated protein kinase (AMPK) and inhibited by mammalian target to rapamycin (mTOR) 29. In human gastric cancer, ULK1 is commonly over-expressed in cancerous tissues and is correlated with patients' T classification as well as cancer relapse 30. Gastrin induces autophagy via activation of the AMPK-ULK1 signaling pathway, which is involved in increased migration and cell survival 31. In MET-amplified gastric cancer, the MET-mTOR-ULK1 cascade is responsible for MET tyrosine kinase inhibitors (MET-TKIs)-mediated autophagy 32. For these patients, MET-TKIs combined with autophagy inhibitors may be a promising therapeutic choice 32. MiR-1262 adds a new layer of ULK1 regulation in gastric cancer.

In summary, to our knowledge, this study is the first to elucidate the crucial involvement of miR-1262 and its functional rs12740674 genetic variation in GCA. These results underline the complexity of genetic regulation of miRNAs during carcinogenesis. Several limitations of the study exist, such as inherent selection bias and the limited sample size. In view of our results showing attenuated miR-1262 expression in human GCA clinical specimens, we hypothesize that miR-1262 may be a potential diagnostic and/or therapeutic target for GCA.

## Supplementary Material

Supplementary figures and tables.

## Figures and Tables

**Table 1:** Genotype frequencies of *miR-1262* rs12740674 genetic polymorphism among cases and controls and their association with GCA risk

Studies	Genotypes	Cases No. (%)	Controls No. (%)	OR^#^ (95% CI)	*P*^#^
		*n* = 584	*n* = 568		
Shandong set	CC	425 (72.8)	371 (65.3)	1.00 (Reference)	
	CT	140 (24.0)	184 (32.4)	0.67 (0.51-0.89)	0.005
	TT	19 (3.2)	13 (2.3)	1.10 (0.75-1.60)	0.627
	CT+TT	159 (27.2)	197 (34.7)	0.71 (0.55-0.92)	0.011
					
		*n* = 440	*n* = 550		
Jiangsu set	CC	324 (73.6)	362 (65.8)	1.00 (Reference)	
	CT	106 (24.1)	175 (31.8)	0.67 (0.50-0.90)	0.008
	TT	10 (2.3)	13 (2.4)	0.84 (0.54-1.29)	0.426
	CT+TT	116 (26.4)	188 (34.2)	0.67 (0.50-0.89)	0.006
					
		*n* = 1024	*n* = 1118		
	CC	749 (73.1)	733 (65.6)	1.00 (Reference)	
Combined	CT	246 (24.1)	359 (32.1)	0.67 (0.55-0.82)	1.1×10^-4^
	TT	29 (2.8)	26 (2.3)	0.98 (0.74-1.30)	0.890
	CT+TT	275 (26.9)	385 (34.4)	0.69 (0.57-0.84)	2.1×10^-4^

Note: GCA, gastric cardia adenocarcinoma; NC, not calculated; OR, odds ratio; CI, confidence interval. #Data were calculated by logistic regression with adjustment for age, sex, smoking and drinking status.

**Table 2:** Risk of GCA associated with *miR-1262* rs12740674 genetic polymorphism by age, sex, smoking, and drinking status

Variables	*miR-1262* rs12740674				
	CC^*^	CT+TT^#^	OR^#^ (95% CI)	*P*	*P*_interaction_
**Age (year)**					0.039
≤66	400/364	140/194	0.60 (0.46-0.79)	2.6×10^-4^	
>66	349/369	135/191	0.79 (0.60-1.04)	0.097	
**Sex**					0.173
Male	622/605	239/319	0.74 (0.60-0.92)	0.006	
Female	127/128	36/66	0.46 (0.27-0.78)	0.004	
**Smoking status**					0.041
Nonsmoker	309/553	132/308	0.58 (0.45-0.74)	1.4×10^-5^	
Smoker	359/180	143/77	0.96 (0.66-1.39)	0.817	
**Alcohol drinking**					0.018
No	435/462	152/268	0.59 (0.47-0.75)	1.6×10^-5^	
Yes	314/271	123/117	0.97 (0.65-1.44)	0.884	

*Number of case patients with genotype/number of control subjects with genotype(s). #Data were calculated by logistic regression, adjusted for sex, age, smoking, and drinking status, where it was appropriate.

## Abbreviations

GCA: gastric cardia carcinoma
miRNA: microRNA
GWAS: genome-wide association studies
SNP: single nucleotide polymorphisms
TCGA: The Cancer Genome Atlas
ENCODE: Encyclopedia of DNA Elements
STAD: stomach adenocarcinoma
AP-1: activating protein 1
AMPK: adenosine monophosphate-activated protein kinase
mTOR: mammalian target to rapamycin
Met-TKIs: Met tyrosine kinase inhibitors
