# Supplementary material for: miR-1262 suppresses gastric cardia adenocarcinoma via targeting oncogene ULK1
Source: J Cancer. 2021 Jan 1;12(4):1231–9. doi: 10.7150/jca.46971 (PMC7797638; doi:10.7150/jca.46971)
Supplement: Supplementary file 1 — Supplementary figures and tables. [file jcav12p1231s1.pdf]

Table S1. Distribution of selected characteristics among GCA cases and controls

Variable	Shandong set (Discovery set)			Jiangsu set (Validation set)		
	Cases	Controls	$P^{\dagger}$	Cases	Controls	$P^{\dagger}$
	No. (%)	No. (%)		No. (%)	No. (%)	
	n = 584	n = 568		n = 440	n = 500	
Age (year)			0.458			0.320
≤66	314(53.8)	293(51.6)		226(51.4)	265(48.2)	
>66	270(46.2)	275(48.4)		214(48.6)	285(51.8)	
Sex			0.598			0.512
Male	494(84.6)	474(83.5)		367(834)	450(81.8)	
Female	90(15.4)	94(16.5)		73(16.6)	100(18.2)	
Smoking status			<0.001			<0.001
No	309(52.9)	449(79.0)		213(48.4)	412(74.9)	
Yes	275(47.1)	119(21.0)		227(51.6)	138(25.1)	
Drinking status			0.006			0.005
No	349(59.8)	384(97.6)		238(54.1)	346(62.9)	
Yes	235(40.2)	184(32.4)		202(45.9)	204(37.1)	
Disease stage						
I	43(7.4)			35(8.0)		
II	115(19.7)			90(20.4)		
III	269(46.0)			202(45.9)		
IV	157(26.9)			113(25.7)		

Note: GCA, gastric cardia adenocarcinoma.
†Two-sided χ^2 test.

Table S2. Associations between *miR-1262* rs12740674 genetic polymorphism and GCA risk stratified by disease stages

Stages	Genotypes	Cases No. (%)	Controls No. (%)	OR† (95% CI)	$P^{\dagger}$
		n = 78	*n* = 1118		
	CC	54(69.2)	733(65.6)	1.00(Reference)	
I	CT	22(28.2)	359(32.1)	1.28(0.46-3.57)	0.642
	TT	2(2.6)	26(2.3)	N.C.	N.C.
		n = 205	*n* = 1118		
	CC	155(75.6)	733(65.6)	1.00(Reference)	
II	CT	39(19.0)	359(32.1)	0.52(0.26-1.03)	0.060
	TT	11(5.4)	26(2.3)	1.21(0.48-3.03)	0.682
		n = 471	*n* = 1118		
	CC	337(71.5)	733(65.6)	1.00(Reference)	
III	CT	124(26.4)	359(32.1)	0.61(0.40-0.94)	0.026
	TT	10(2.1)	26(2.3)	0.95(0.49-1.84)	0.870
		n = 270	*n* = 1118		
	CC	203(75.2)	733(65.6)	1.00(Reference)	
IV	CT	61(22.6)	359(32.1)	0.75 (0.42-1.34)	0.333
	TT	6(2.2)	385(34.4)	0.77(0.36-1.66)	0.502

Note: GCA, gastric cardia adenocarcinoma; N.C., not calculated; OR, odds ratio; CI, confidence interval.
†Data were calculated by logistic regression with adjustment for age, sex, smoking and drinking status.

Table S3. Oligonucleotides used in the current study

#	**Oligonucleotide Sequences (5'→3')**
rs12740674 Sequenom Primers	
PCR primers-F	ACGTTGGATGTGACATCCTGATGAGTCAGC
PCR primers-R	ACGTTGGATGGACTAATCCCCATCTCTCTC
UEP_SEQ primer	CCAAATAACTCAGGGTCA
EXT1_SEQ primer	CCAAATAACTCAGGGTCAC
EXT2_SEQ primer	CCAAATAACTCAGGGTCAT
ULK1 siRNA duplexes	
siULK1-1	UCAUCACCCUUUCCUCGAUTT/AUCGAGGAAAGGGUGAUGATT
siULK1-2	CCUGUGACACAGACGACUUTT/AAGUCGUCUGUGUCACAGGTT
siULK1-3	GCAGAACUACCAGCGCAUUTT/AAUGCGCUGGUAGUUCUGCTT
ULK1 qRT-PCR primers	
ULK1-qF	GGCAAGTTCGAGTTCTCCCG
ULK1-qR	CGACCTCCAAATCGTGCTTCT
β-actin qRT-PCR primers	
β-actin-qF	GGCGGCACCACCATGTACCCT
β-actin-qR	AGGGGCCGGACTCGTCATACT
miR-1262 reporter gene primers	
p-491F	CGGGGTACCGTCATCTCAGAAACCAAGTT
p-491R	CCGCTCGAGCTTCCTTCCTATTTTCAGGT
ULK1 3'-UTR reporter gene constructs	
ULK1-3'-UTR-F	CCGCTCGAGCTGTGCCCAGGAAGAGCCTG (XhoI)
ULK1-3'-UTR-R	CTAGAAGCTTGGCATGTGTCTGCATATGTG (HindIII)
Mutagenesis 3'-UTR-F	ACCCAGCTTTGTCAAGACATAGCGCACTTTATGCATATAG
Mutagenesis 3'-UTR-R	CTATATGCATAAAGTGCGCTATGTCTTGACAAAGCTGGGT

Supplementary Figures

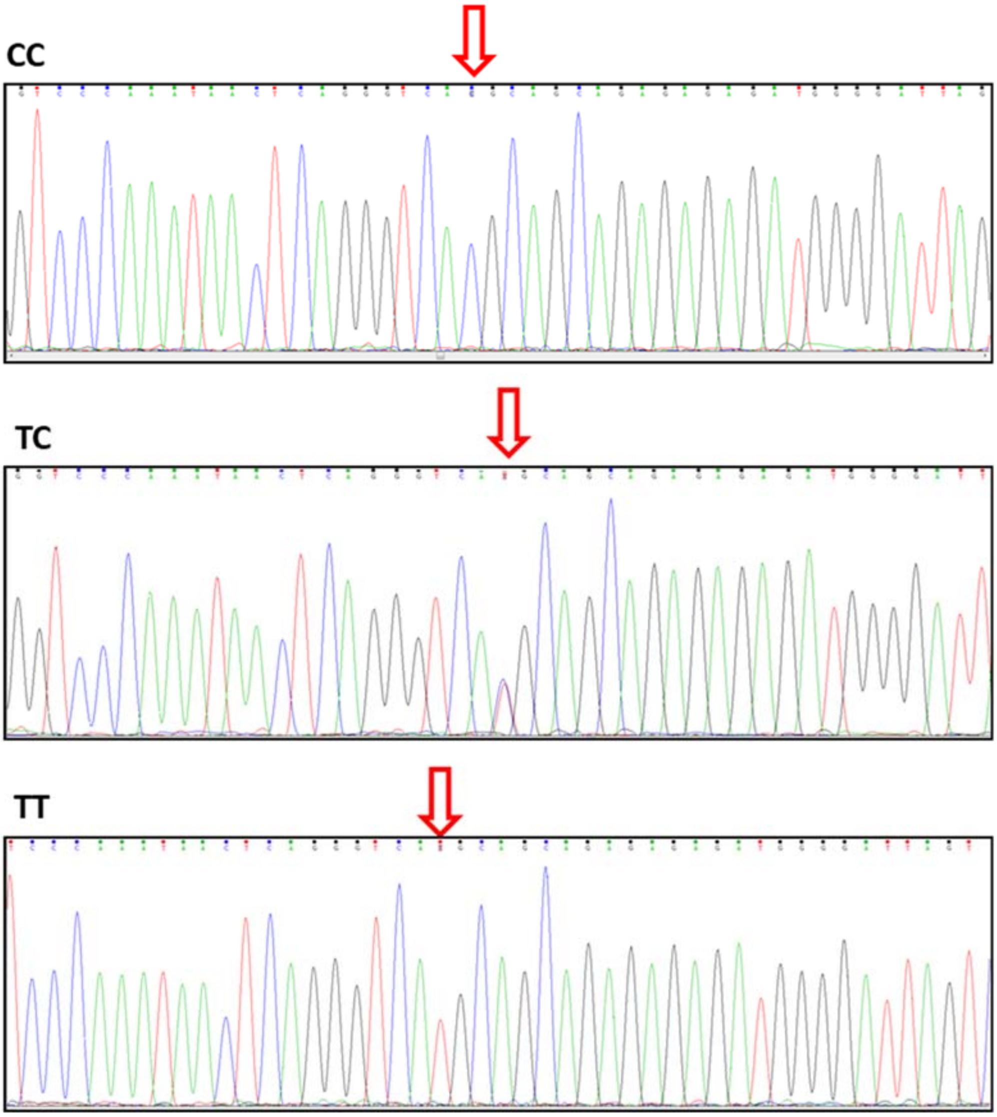

Figure S1. Sanger sequencing of rs12740674 CC, CT and TT genotypes of GCA tissues.

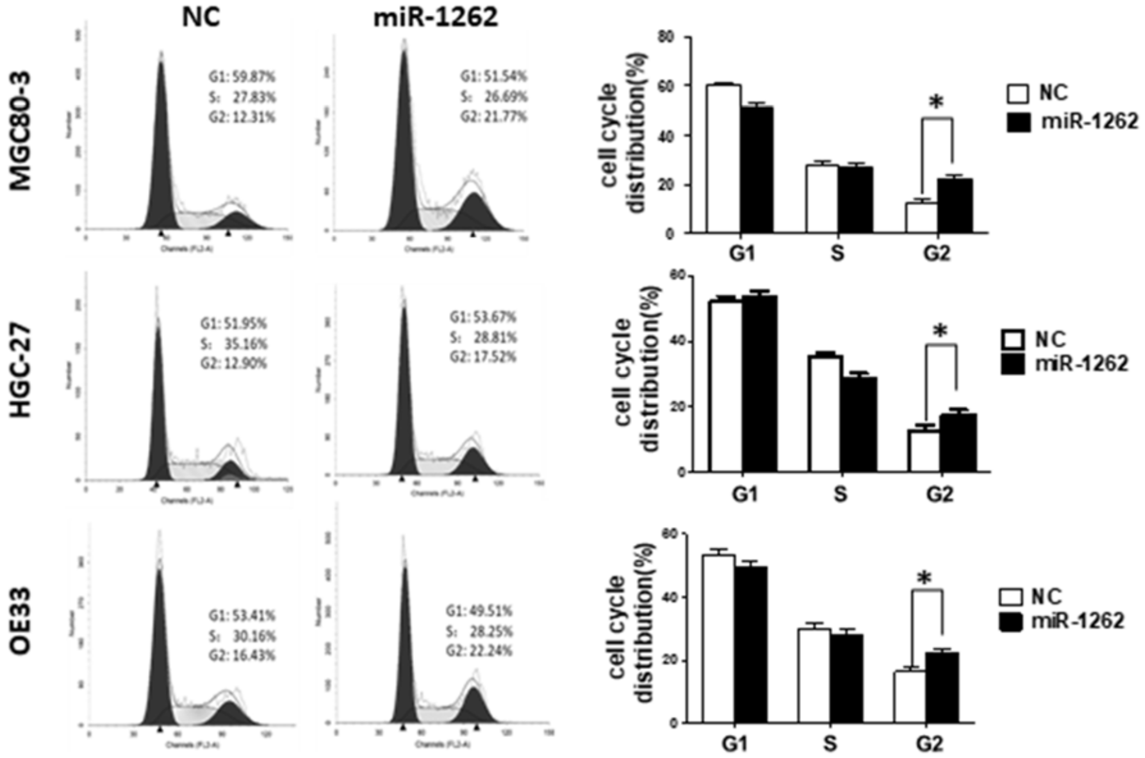

Figure S2. miR-1262 elevates G2/M populations of MGC80-3, HGC-27 and OE33 cells. MGC80-3, HGC-27 and OE33 cells were transfected with miR-1262 mimics or NC RNA, 48 hours after transfection, cells were collected and dyed with PI, detected with the FACS Calibur FCM. **P*< 0.05

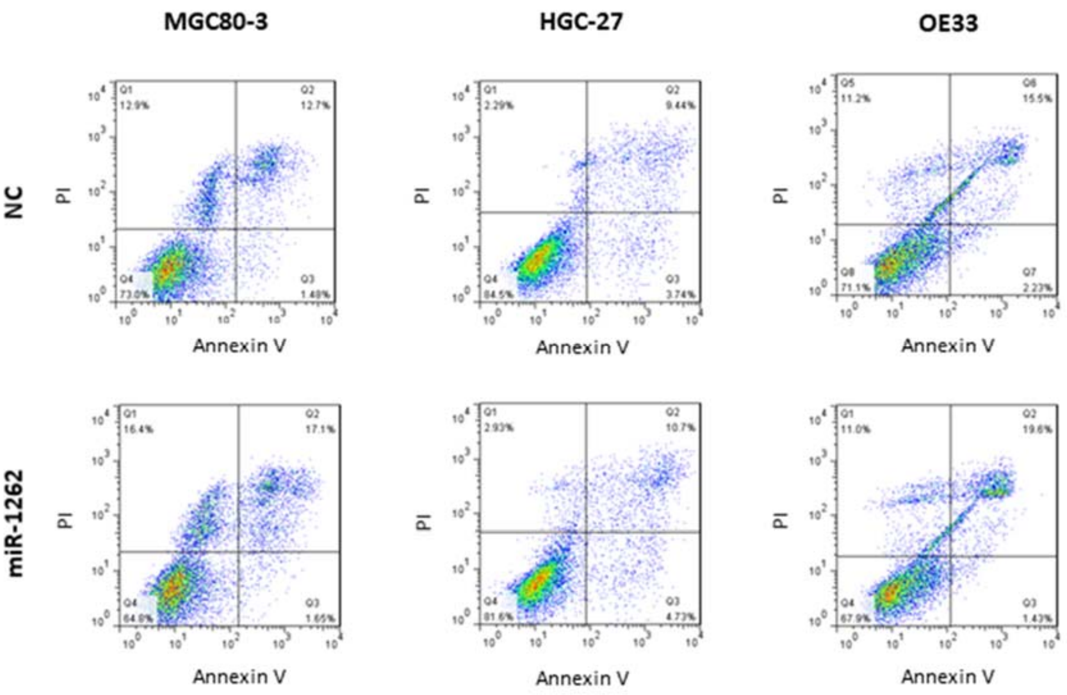

Figure S3. miR-1262 does not influence the apoptosis of MGC80-3, HGC-27 and OE33 cells. MGC80-3, HGC-27 and OE33 cells were transfected with miR-1262 mimics or NC RNA, 48 hours after transfection, cells were collected and determined using the Alexa Fluor 488 annexinV/Dead Cell Apoptosis Kit with FACS Calibur flow cytometer. **P*< 0.05. One of three experiments with similar results is shown.

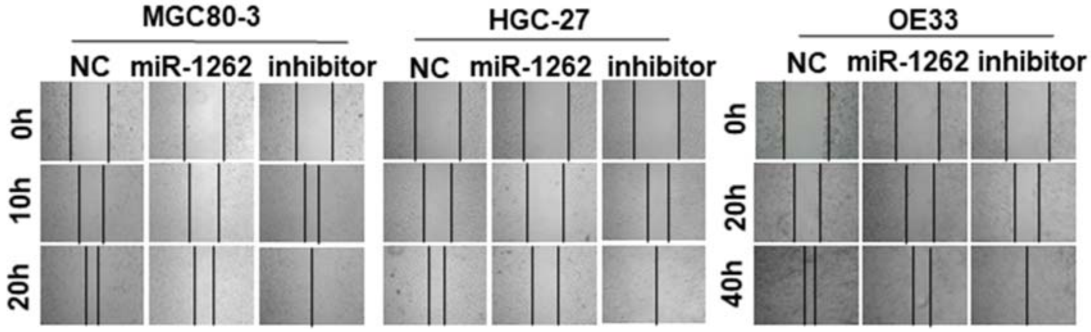

Figure S4. miR-1262 inhibits wound healing in MGC80-3, HGC-27 and OE33 cells. MGC80-3, HGC-27 or OE33 cells were transfected with miR-1262 mimics, miR-1262 inhibitors, or NC RNA, a wound was scratched by a 10μl pipette tip. The average extent of wound closure was measured at different time points. One of three experiments with similar results is shown.

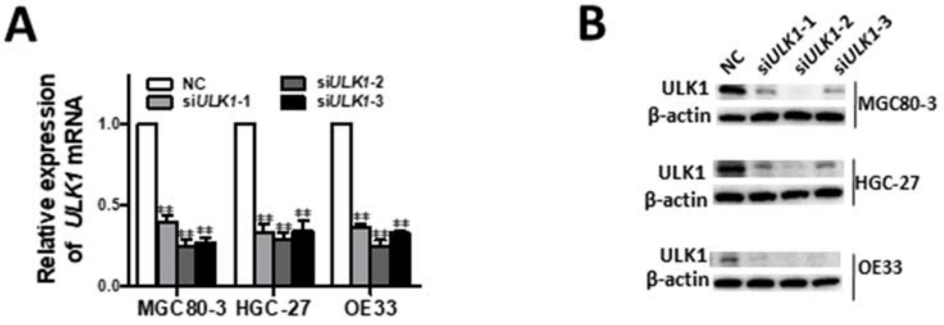

Figure S5. siRNAs targeting to *ULK1* successfully reduced ULK1 expression (A) *ULK1* mRNA expression level detected using qRT-PCR method. (B) ULK1 protein expression level detected using western blotting method. One of three experiments with similar results is shown.

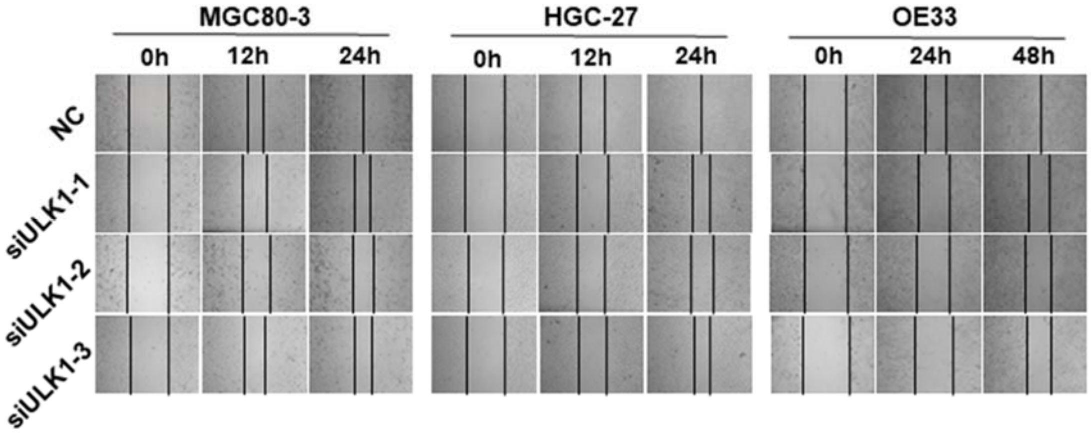

Figure S6. ULK1 siRNAs inhibit wound healing in MGC80-3, HGC-27 and OE33 cells. MGC80-3, HGC-27 or OE33 cells were transfected with*ULK1* siRNAs or NC RNA, a wound was scratched by a 10μl pipette tip. The average extent of wound closure was measured after MGC80-3, HGC-27 or OE33 cells were continued cultured at 37°C. One of three experiments with similar results is shown.
